# Low rate of relapse after twelve-dose multidrug therapy for hansen’s disease: A 20-year cohort study in a brazilian reference center

**DOI:** 10.1371/journal.pntd.0009382

**Published:** 2021-05-03

**Authors:** José A. C. Nery, Anna M. Sales, Mariana A. V. B. Hacker, Milton O. Moraes, Raquel C. Maia, Euzenir N. Sarno, Ximena Illarramendi

**Affiliations:** 1 Souza Araújo Outpatient Clinic, Oswaldo Cruz Institute, Oswaldo Cruz Foundation, Rio de Janeiro, Brazil; 2 Leprosy Laboratory, Oswaldo Cruz Institute, Oswaldo Cruz Foundation, Rio de Janeiro, Brazil; 3 Center for Technological Development in Health, Oswaldo Cruz Foundation, Rio de Janeiro, Brazil; KU Leuven, BELGIUM

## Abstract

The World Health Organization has raised concerns about the increasing number of Hansen disease (HD) relapses worldwide, especially in Brazil, India, and Indonesia that report the highest number of recurrent cases. Relapses are an indicator of MDT effectiveness and can reflect *Mycobacterium leprae* persistence or re-infection. Relapse is also a potential marker for the development or progression of disability. In this research, we studied a large cohort of persons affected by HD treated with full fixed-dose multibacillary (MB) multidrug therapy (MDT) followed for up to 20 years and observed that relapses are a rare event. We estimated the incidence density of relapse in a cohort of patients classified to receive MB regime (bacillary index (BI) > 0), diagnosed between September 1997 and June 2017, and treated with twelve-dose MB-MDT at a HD reference center in Rio de Janeiro, Brazil. We obtained the data from the data management system of the clinic routine service. We linked the selected cases to the dataset of relapses of the national HD data to confirm possible relapse cases diagnosed elsewhere. We diagnosed ten cases of relapse in a cohort of 713 patients followed-up for a mean of 12.1 years. This resulted in an incidence rate of 1.16 relapse cases per 1000 person-year (95% CI = 0.5915–2.076). The accumulated risk was 0.025 in 20 years. The very low risk observed in this cohort of twelve-dose-treated MB patients reinforces the success of the current MDT scheme.

## Author summary

People affected by Hansen disease that have many skin patches or lumps receive twelve-dose multibacillary multidrug therapy. After the fixed-dose regime, they are released from treatment and are considered cured. However, some of these people may have a recurrence of skin lesions or peripheral nerve problems after several years. Clinicians, scientists, and the World Health Organization are concerned about this problem, known as relapse, which seems to be increasing in countries where Hansen disease is common, especially in Brazil, India, and Indonesia. It is important to demonstrate regular proof that the therapy remains effective to treat people affected by Hansen disease. In this study, we showed that the recurrence of signs and symptoms of Hansen disease after the twelve-dose multibacillary treatment is rare. These findings are good evidence of the success of the current multibacillary scheme.

## Introduction

The treatment duration of Hansen disease (HD), a chronic infection caused by *Mycobacterium leprae (M*.*leprae)*, has been progressively reduced as new antibiotics and evidence have emerged. It started with dapsone monotherapy that could be given during the patient’s lifetime. However, the emergence of dapsone resistance in the late 1970´s led to the introduction of multidrug therapy (MDT). Considered highly effective [1], in 1982 a fixed-dose multibacillary (MB)-MDT regime was implemented for 24-monthly-doses, which was later reduced to twelve doses in most endemic countries [2]. Although termination of treatment is based on completion of the recommended regime rather than disappearance of clinical signs and symptoms or bacteriological clearance, this reduction of treatment duration did not appear to impact relapse rates [3].

After more than three decades since MDT implementation, the number of relapses registered is continuously increasing. In 2016, 52 countries reported 2,844 relapses; 51 countries reported 3,192 relapse cases in 2017 [4], and there were 3,361 and 3,893 relapse cases registered in 2018 and 2019, respectively. The highest number of relapses were reported by Brazil (1,698), followed by Indonesia (627) and India (505) [5]. This increase was observed after the World Health Organization (WHO) sentinel relapse network was created to monitor resistance in circulating *M*.*leprae* strains and represent relapses from all types of treatment schemes, pauci and multibacillary, twelve- and 24-fixed doses, and even monotherapy. However, the latest WHO Global HD Strategy 2016–2020 indicates that relapses or drug-resistance are not currently a major public health concern [6]. During the first global surveillance action, samples obtained between 2009 and 2015 from 1,932 patients were tested. Among the 1,143 relapse cases tested, 5.1% showed rifampicin resistance [7].

The relapse rate is an accepted measure of outcome to evaluate MDT efficacy [8]. Therefore, it is essential to get an accurate representation of the relapse rate of the current twelve-dose MB-MDT for the treatment of HD. This is of particular importance to the narrative of discussions regarding the future reduction of the length of HD therapy, which is recommended by the WHO [9]. Yet, few long-term studies have been published regarding the incidence rate (IR) of relapse in patients treated exclusively with fixed twelve-dose MDT [10–12]. By assessing the incidence of relapse in a cohort of MB-MDT-treated patients in a HD reference center, the present study brings new evidence that relapse after the twelve-dose MB-MDT scheme is not a significant problem in HD.

## Methods

### Ethics statement

Oswaldo Cruz Foundation—Fiocruz/IOC Research Ethics committee approved the protocol registered under the number (CAAE) 86568618.1.0000.5248 in 10/04/2018. The institutional review board allowed informed consent exemption because the data was obtained from the *Sistema ASA* and was anonymized for analysis. Exemption was also requested due to the hurdle of obtaining written consent from patients in a retrospective study.

This is an observational retrospective cohort study of patients selected from the routine healthcare activities at the Souza Araújo Outpatient Clinic (ASA), Fiocruz, a HD reference centre in Rio de Janeiro, Brazil. All patients selected were diagnosed, completed full twelve-dose MB-MDT scheme, and followed-up at ASA. The starting date for follow-up was considered as the date of each patient’s release from treatment (RFT), and the last date of follow-up was December 2019 (or the date of relapse diagnosis or death during follow-up). For elderly patients, follow-up was censored according to life expectancy, with highest value given according to the age of the eldest patient (90 years) in the cohort. In summary, patients with positive bacillary index (BI) diagnosed between September 1997 and June 2017 and treated with fixed twelve-dose MB-MDT were selected, and followed-up for at least one year until December 2019.

Although WHO definitions for MB operational classification have been adjusted throughout the past 20 years (according to different categories, such as BI and number of skin and/or nerve lesions) [2], in the ASA clinic, all patients with BI greater than zero at diagnosis, regardless of the number of lesions or the Ridley and Jopling classification [13], receive MB-MDT. The latter classification is used to identify the clinical and immunopathological forms of the patients. Briefly, the patients are classified into two polar forms, tuberculoid or lepromatous leprosy, or three intermediate forms, borderline-tuberculoid, borderline-borderline and borderline-lepromatous. The patients may also be classified with the indeterminate form, an early stage of HD in which the immune response cannot be defined. The patients in the tuberculoid pole have an effective cellular immune response, which contains the infection, thus developing few localized lesion. Patients on the lepromatous spectra have an ineffective immune response, and proliferation of skin lesions with large concentration of bacilli.

The patients receive the twelve-dose course of standard MB-MDT with directly observed monthly 600 mg rifampicin, 300 mg clofazimine and 100 mg dapsone, and daily self-administered 50 mg clofazimine and 100mg dapsone. Patients with contra-indications or adverse effects to any of these drugs receive alternative MDT according to the Brazilian Ministry of Health (MoH) guidelines [14]. At our center, as part of the routine, the patients are monitored annually after RFT for up to ten years. Besides, the patients are advised to return to the clinic whenever they recognize new or worsening of any dermatological or neurological symptoms or signs. Before diagnosis and after MDT, at their annual appointment or when necessary, the patients are clinically examined by a dermatologist and a neurologist and are evaluated by the physiotherapist to determine the disability grade (DG). The DG is classified according to the Brazilian MoH [14] criteria, as follows: 0—no eyes, hands or feet problems due to HD; 1—sensory function reduction of the eyes, hands and/or feet; and 2—visible deformity or damage in the eyes, hands and/or feet.

Slit-skin smears samples are obtained from 4–6 sites (including the skin lesion, if present). The samples are stained using the Ziehl-Nielsen to calculate the BI, which is the logarithmic product of the number of acid-resistant bacilli observed in 100 or 25 (if > 10 bacilli/field are identified) microscopy fields [15]. The Logarithmic Biopsy Index (LBI) is calculated during the histopathological analysis of the biopsy sample to identify acid-resistant bacilli by modified Fite-Faraco staining, according to Ridley & Jopling [13]. It is the result of the product of the bacterial index in the granuloma and the fraction of the dermis occupied by the granuloma. It has a maximum value of 6.0. Both the BI and the LBI are important to measure the bacterial load of the patient.

For the present study, relapse definition was adapted from the WHO Scientific Working Group on Chemotherapy of Leprosy–THELEP [16]. The patients were diagnosed with a relapse if, after finishing a complete full course of twelve-dose MB-MDT, they developed new HD skin lesions and/or had reactivation of old lesions, and were non-respondent to anti-reactional treatment, or if they had BI increase during surveillance after RFT. Patients could also have peripheral nerve dysfunction.

Patient data were obtained from the ASA data management system (*Sistema ASA*). In addition, the clinical files of the relapse cases were reviewed. The methods of selection of the study sample from the data system, that is, the codes and algorithm used to identify subjects, as well as the algorithms used to classify exposures, outcomes, confounders, and effect modifiers as recommended by the *REporting of studies Conducted using Observational Routinely-collected health Data* (RECORD) statement [17] can be supplied upon demand.

Although patients are instructed to return to the clinic in case of any new signs or symptoms, some of them might be diagnosed with relapse at other health centers and, therefore, not return to the clinic. In order to capture possible relapse cases diagnosed elsewhere due to change of address or for other reasons, the patients registered in the ASA data management system were looked for among the relapse cases registered in the National notifiable disease registry SINAN (from Portuguese, “Sistema de Informação de Agravos de Notificação”) from 2001 and January 2021. The databases were linked using the Reclink program [18] employing a probabilistic method that compares the patient’s name, date of birth, sex, and the mother’s name. The relapse cases registered by ASA were excluded from the dataset. The patient was considered free of relapse if his/her name was absent in the linked file.

The IR considered the period that every one of the cohorts was exposed to the risk of relapse given by the follow-up time calculated for each patient. The follow-up time was censored so that the added value of age and time of follow-up was below or equal to 90, adjusted to the life expectancy at the year of birth of the eldest patient in the cohort (according to the Brazilian Institute of Geography and Statistics https://www.ibge.gov.br/estatisticas/sociais/populacao/9126-tabuas-completas-de-mortalidade.html). The incidence of relapse in person-year (py) was calculated by the quotient between the number of relapse cases and the total person-time of the cohort. The accumulated risk curve over the years of follow-up was calculated using the Kaplan-Meier method. Spline smoothing was used to show the curve of annual BI mean from relapse and non-relapse cases. IBM SPSS Statistics for Windows, Version 25.0. Armonk, NY: IBM Corp was used for statistical analysis.

## Results

**Fig 1:**
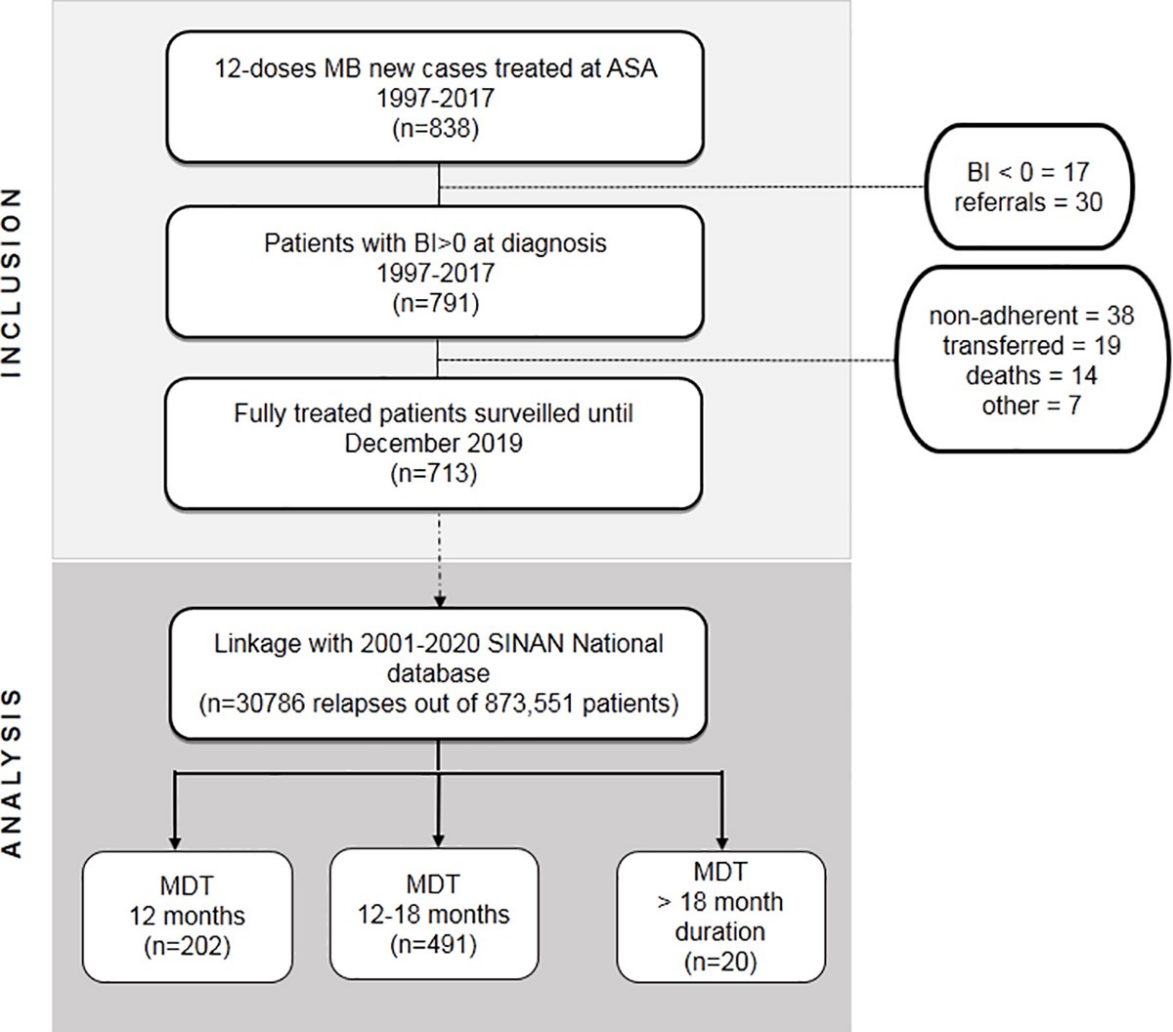
Flow-diagram of the Fiocruz Hansen disease Reference Center Cohort. The inclusion criteria considered patients who exhibited a BI higher than 0 at diagnosis, were treated with the 12-dose MB-MDT scheme between 1998–2018, and who were followed-up at the Souza Araujo Outpatient Clinic after release from treatment. Patients were followed-up at least annually (except for those censored because of age or death). Linkage of the clinic data with the National HD registry SINAN database was performed to retrieve any relapse cases not diagnosed at the clinic due to change of address. Then, the patients in the follow-up cohort were grouped according to the length of the 12-dose MB-MDT treatment. BI, bacillary index; MB, multibacillary; MDT, multidrug therapy.

Out of the 838 people affected by HD admitted at the clinic for treatment with twelve-dose MDT, 713 patients (BI > 0, without previous treatment) were considered cured and released from treatment between September 1998 and June 2018 (Fig 1).

The majority of patients were male (71.4%), with ages ranging from 4 to 90 years, and a mean age of 40.2 ± 17.2 years (Table 1). A large proportion of patients was classified with the lepromatous leprosy (LL) clinical form but most patients (58.7%) had between 6–20 skin lesions at time of diagnosis. Although a large proportion of patients had a high BI at diagnosis, at RFT already 65.6% of the patients had BI below 3.5.

**Table 1:** Epidemiological and clinical characteristics at diagnosis of the 713-patient cohort treated with multibacillary multidrug therapy at Souza Araújo Outpatient Clinic, Fiocruz, during 1997–2017.

Characteristic	Number of patients (valid %)	
**Sex**		
Male	509 (71.4)	
Female	204 (28.6)	
**Educational level (years)**		
0—informal	80 (11.4)	
<9	357 (50.8)	
9–11	134 (19.1)	
>11	131 (18.7)	
Not available	11	
**Clinical Form**		
Lepromatous leprosy	330 (46.3)	
Borderline-Lepromatous	237 (33.2)	
Borderline -Borderline	141 (19.7)	
Borderline-Tuberculoid	3 (0.4)	
Indeterminate	2 (0.3)	
**Number of skin lesions**		
Single lesion	21 (3.6)	
2–5 lesions	46 (7.8)	
6–10 lesions	50 (8.5)	
11–20 lesions	294 (50.2)	
More than 20 lesions	175 (29.9)	
Not available	127	
Treatment duration (months)*		
12	202 (28.3)	
13–18	491 (68.9)	
> 18	20 (2.8)	
**Disability grade**	**Initial**	**RFT**
0 (no disability)	362 (56.5)	331 (55.9)
1 (anaesthesia/paresis)	180 (28.1)	187 (31.6)
2 (deformity/ulcer)	99 (15.4)	74 (12.5)
Not available	72	121
**Bacillary index**	**Initial**	**RFT (n = 531)**
0	0 (0.0)	98 (18.4)
0.05–3.38	343 (48.5)	251 (47.2)
≥3.5	367 (51.5)	183 (34.4)
Median	3.50	2.50
Minimum, maximum	0.16, 6.00	0.0, 5.90
First and third quartiles	1.75, 4.25	0.50, 3.76

*All patients received a twelve-dose MB-MDT scheme, but some were non-compliant with the monthly schedule or had to delay treatment due to adverse effect. Treatment duration was calculated using the date the patient received his/her first supervised dose until he/she received the last supervised dose, regardless the time taken to restart the treatment in case of non-compliance.

RFT = release from treatment

Most patients underwent regular treatment according to the Brazilian Ministry of Health [14] recommendations, i.e. they received 12 doses in 12–18 months, but 2.8% had irregular treatment lasting more than 18 months. These were patients that interrupted treatment at the beginning after 1–2 doses and started over one or two years later. Twenty-one patients received alternative treatment due to adverse effects. In 20 patients, dapsone was substituted with 100 mg/day clofazimine (n = 12), ofloxacin (n = 7) or minocycline (n = 1). One patient had both dapsone and rifampicin substituted for ofloxacin and minocycline and received an additional 50 mg/day clofazimine.

The patients were followed for approximately 12 years on average, totaling 8,587.31 py. Ten patients were diagnosed with relapse during follow-up at ASA. No additional relapse cases were identified after the linkage of the ASA dataset and the 30,786 relapse cases registered in the National SINAN database. These ten patients resulted in a relapse IR of 1.16 per 1000 py (95% CI = 0.5915–2.076).

The relapse cases were all adults and received standard MB-MDT for 12–13 months (Table 2). Seven out of these patients had active skin lesions that were biopsied and whole bacilli were found in 4 of the samples. All patients diagnosed with a relapse presented the *M*.*leprae* wild type strain without any directly known resistance mutations to rifampicin, dapsone, or fluoroquinolones.

**Table 2:** Demographic and clinical characteristics of the ten patients diagnosed with Hansen disease relapse.

			First diagnosis						Relapse			
			Number of skin lesions	Type of lesion	R&J classification [13]	DG		MDT duration (months)	Number of skin lesions	Type of lesion	R&J classification [13]	DG
	Sex	Age				**Before**	**End**					
1	M	31	>20	tubercles, infiltration	LL	1	1	12	0	-	ND	1
2	F	46	>20	plaques	BL	1	0	12	0	-	ND	2
3	M	37	>20	plaques, tubercles	LL	0	0	13	>20	plaques	BL	0
4	F	40	>20	nodules, infiltration	LL	1	1	12	11–20	plaques	BL	NA
5	M	26	11–20	tubercles, infiltration	LL	0	0	12	>20	plaques, infiltration	LL	0
6	M	18	6–10	macules	BB	0	0	12	6–10	macules	BB	0
7	M	23	11–20	plaques	BL	1	1	13	>20	tubercles, infiltration	LL	1
8	M	24	11–20	plaques, infiltration	LL	0	0	12	>20	plaques, tubercles	LL	2
9	M	53	>20	tubercles	LL	0	0	12	>20	tubercles, infiltration	LL	0
10	M	25	>20	plaques	BB	0	0	12	>20	plaques, tubercles	BL	0

m = male, f = female, LL = lepromatous leprosy, BL = borderline leprosy, ND = not defined (no skin lesions for confirmation by biopsy), MDT = multidrug therapy, R&J = Ridley and Jopling, DG = disability grade, NA = not available.

**Fig 2:**
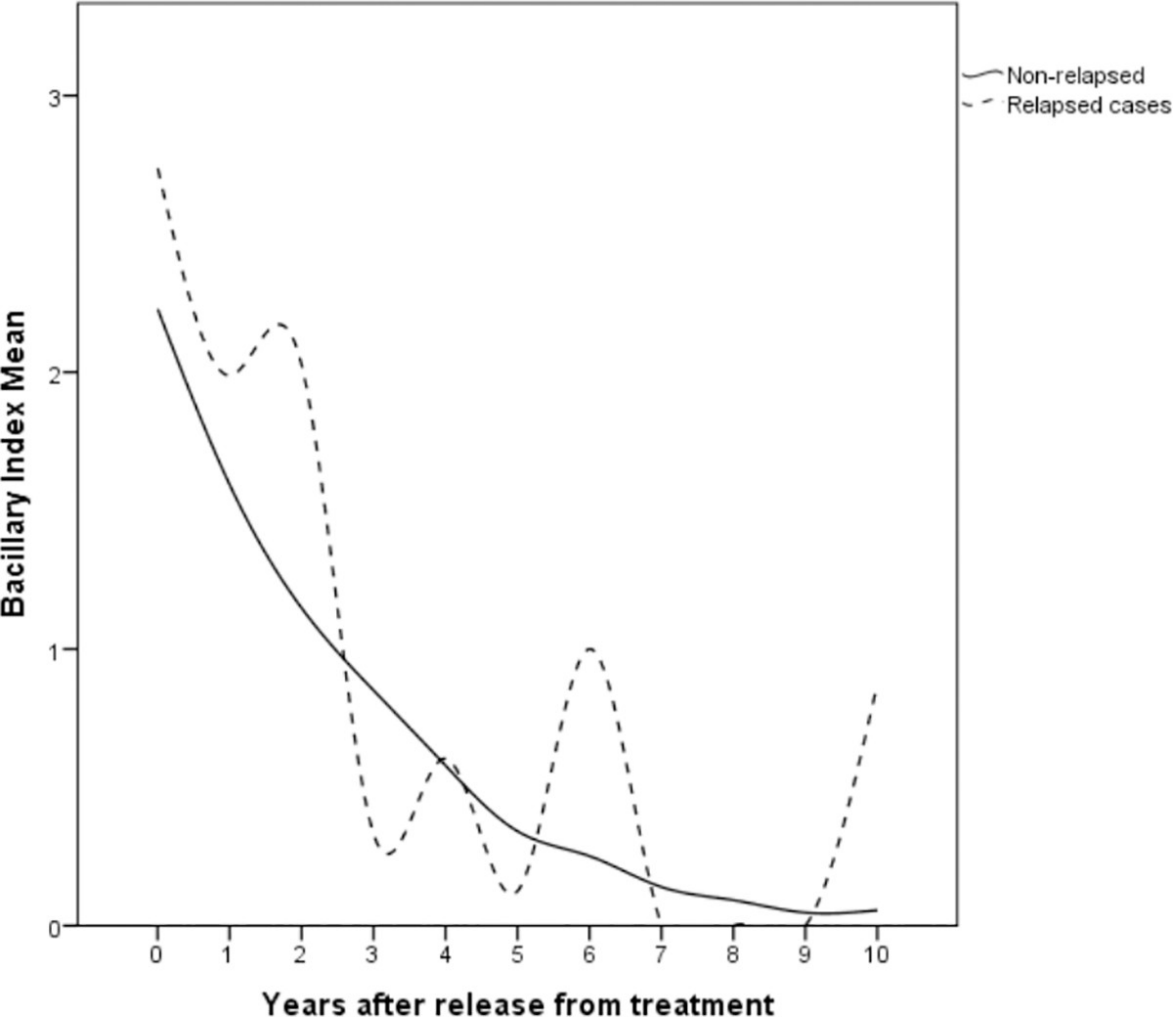
Spline curve of the annual bacillary index (BI) mean values in 12-dose MB-MDT Hansen disease patients. Progressive decrease of the BI obtained from slit-skin smear samples of non-relapse cases during the first years after release from treatment (full line). Observe that positive BI values remain long after release from treatment. Relapse cases (dotted line), however, show greater variability, possibly due to the small number, and display BI increases before relapse.

Nine patients with relapse reached zero or near zero BI after an average of 9.3 years of follow-up (Table 3). Interestingly, median BI at RFT was 3.66 (minimum of 1.00 and maximum of 4.16) and at relapse, median BI was 1.25 (minimum = 0.25 and maximum of 5.00). Median years to zero BI was 6.9 years (first quartile = 4.1 and third quartile = 7.33) for relapse cases. A steady BI reduction was observed in non-relapses (Fig 2) that took on average 2.0 years (first quartile = 0.83 and third quartile = 4.67 years) to attain zero BI.

**Table 3:** Laboratory characteristics of the ten patients diagnosed with Hansen disease relapse.

	First diagnosis–Bacterial load				Surveillance			Relapse–Bacterial load			
	BI		LBI		Number of years until BI~0	Lowest BI	Years from RFT to relapse	BI	LBI	
	Before MDT	End MDT	Before MDT	End MDT				Before MDT	End MDT	Before MDT	Observations
1	4.16	3.83	4.85	2.00	4.1	0.75	5.4	1.25	0.75	NE	ENL at 11th dose
2	4.00	1.00	3.50	NA	3.5	0.00	5.6	0.50	0.00	0.00	RR at 12th dose
3	4.00	3.66	5.85	2.30	2.1	0.00	11.6	2.50	2.00	4.50	no reaction
4	4.00	3.66	4.80	3.60	6.9	0.50	13.2	2.25	NA	3.50	ENL at diagnosis
5	3.83	3.75	5.80	3.50	7.3	0.00	8.1	0.25	3.75	5.85	no reaction
6	2.83	3.66	3.60	2.30	8.6	0.00	9.4	0.50	0.00	1.60	neuritis at 11th dose
7	2.16	2.83	4.50	2.50	6.4	0.00	14.9	5.00	4.00	3.50	EM at 1st dose
8	4.00	4.16	3.50	3.50	9.2	0.00	10.5	2.50	0.00	4.50	ENL at 1st dose
9	3.00	3.·50	5.90	3.50	*		3.1	4.00	1.75	3.85	ENL at 2nd dose was treated w/minocycline + history of chronic alcoholism
10	4.00	2.75	4.6	ND	7.0	1	10.5	1.25	1	3.5	Insulin-dependent diabetes, ENL during first treatment.

BI = baciloscopic index, LBI = logarithmic biopsy index, MDT = multidrug therapy, DG = disability grade, ENL = *erythema nodosum leprosum*, RR = reversal reaction, EM = *erythema multiforme*, NA = not available, NE = not evaluated because there was no skin lesion.

*lowest BI was 3.50 at 1.7 years after RFT, and maintained 4.0 thereafter

**Fig 3:**
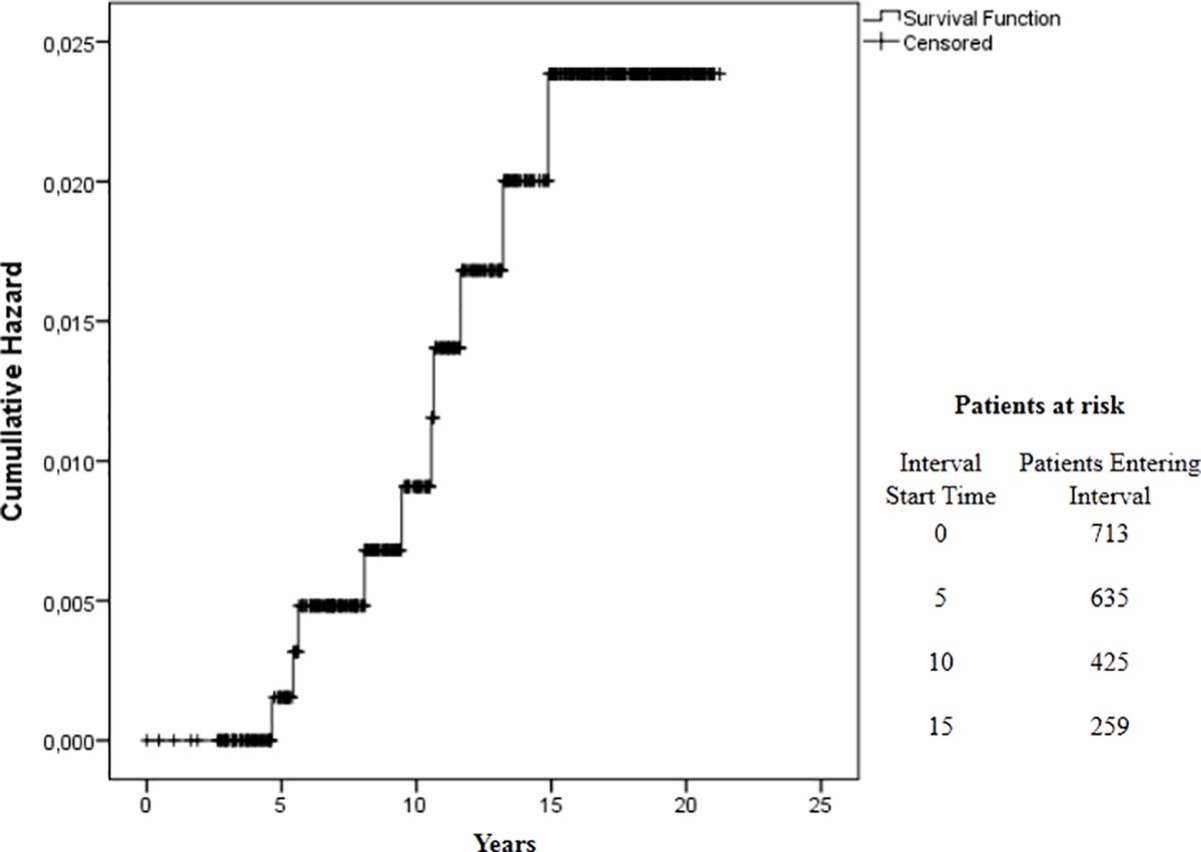
Kaplan-Meier Cumulative Risk of relapse in 713 patients treated with 12-dose MB-MDT. The survival curve shows the proportion of people affected by Hansen disease without relapse according to the period of follow-up. Y-axis of the curve is the cumulative probability of relapse. The X-axis represents the survival duration. The length of the horizontal lines is the interval terminated by the occurrence of relapse events and the heights between horizontal lines illustrate the change in the cumulative probability.

Although high bacillary load has been considered as a risk factor for relapse, in this cohort, out of the 182 patients with BI ≥ 3.50 at RFT, only 7 (3.9%) had relapse. In summary, a very low risk of relapse (accumulated risk of 0.025 in 20 years) was observed in this cohort during the study period (Fig 3).

## Discussion

In the present cohort of patients affected by HD treated with twelve-dose MB-MDT, the relapse IR was low (1.16 per 1000 py (95% CI = 0.5915–2.076)). The period of follow-up was long and variable, because of censoring and loss to follow-up. Thus, the incidence density in this cohort allowed an accurate risk estimate for presenting the outcome of interest [19], in this case, relapse.

The present is the largest and longest single-centre cohort study of relapse in people affected by HD treated exclusively with twelve-dose MB-MDT. A previous study of 730 patients followed-up for a period ranging from 9 months to 10 years reported relapse in 13 (1.8%) patients [20]. Although we found a similar proportion (1.4%) of relapse, that number might be overestimated. Relapse as early as 9 months after RFT is probably a sign of failure or treatment insufficiency, rather than relapse, which occurs later after fixed-dose MB-MDT. In addition, the proportion of relapse cases is a less informative measure of the situation. The IR of HD relapse has been estimated in a few other cohort studies that included patients treated with twelve-dose MB-MDT, but followed-up for shorter periods than the present study. These studies have shown an IR ranging from 0.52/1000 py after a mean of 6.4 years follow-up/patient [12], 1.97/100 py in 162 patients followed for up to 8 years [11], to 7.5/1000 py after up to 7 years of follow-up [10]. Poojabylaiah *et al*. [21] observed no relapses in 41 cases, but indicated the patients were not followed-up for a sufficient period of time after RFT. In another study, the enhancement of the twelve-dose MDT with monthly minocycline and ofloxacin did not reduce the rate of relapse. The authors reported an IR of 0.05/100 py in a cohort of 70 patients that was followed up for 10 years [22]. These large differences between IR may be explained by the diverse criteria used to include patients, define relapse, the period of follow-up, and the levels of endemicity of the different settings.

HD diagnosis is still a challenge because of its similarity with many other skin and peripheral nerve diseases. But the definition of relapse is even more challenging due to the frequent reactions and neurological complications that may occur after RFT. Furthermore, the definition of cure is also controversial in HD, because patients can be released from MDT with a still considerable bacterial load or with apparently active lesions [23]. Many criteria for defining relapse have been proposed and used by national HD programs and researchers [8,15,24]. Deepak & Gazzoli [25] described in a multicenter study conducted in Bangladesh, China, India, the Comoros Islands, Ghana, and Mozambique, the difficulties of using routine data for reporting relapses. The major challenge was the lack of uniformity in the criteria and definition of relapses. Similar difficulties were observed with the use of routine data for the present study. Still, the increase in BI after zeroing, independent of the number of years involved to reach 0, appears to be the best possible way to define relapse. Thus, regular monitoring of the BI to detect an increase could be helpful to diagnose a relapse. However, slit-skin smears are rarely collected in primary care settings where most of the people affected by HD are treated and followed-up.

The time to relapse is also controversial. There is a lack of consensus regarding the adequate time after RFT to consider a case with active lesions and/or positive BI as a relapse. It is important to remember, that the presence of new active lesions after RFT is the standard for defining both, relapse and reactions [14]. In the cohort studied here, the number of years to relapse was variable, but all cases were diagnosed more than 4 years after RFT. Gonçalves *et al*. [26] reported 37 relapses diagnosed in patients that received 10–19 doses of MDT in an average of 7.4 ± 5.3 years, with a minimum of 0.06 years after RFT. Other authors have reported relapse times of less than 5 years after RFT [10,25]. It is worth mentioning that the presence of active skin lesions and positive BI are known to persist after termination of MB-MDT regardless of the BI value at diagnosis [12,23] and may represent reactions. On other occasions, disease reactivation soon after RFT could be due to treatment insufficiency or, more rarely, antibiotic resistance [24]. Therefore, health professionals should exclude reactions or treatment insufficiency when considering relapse at such an early stage after RFT.

Patients who received irregular treatment, *i*.*e*. took more than 12 months to complete MDT or had a modified regimen, could be expected to have higher relapse incidence. However, all of the relapse cases in this cohort used regular MDT and were RFT at 12–13 months after starting MDT. Similarly, Sapkota *et al*. [27] reported no cases of relapse in 36 patients with positive BI treated with modified MDT after up to 10 years of retrospective follow-up. One of the relapses in this cohort received additional minocycline to treat ENL, which is one of the antibiotics used as a substitute for dapsone [14]. Interestingly, this patient had a steady bacillary load and maintained a high bacillary load throughout the first 4 years of follow-up indicating that patients may vary the BI clearance rate, but steady or even very slow decrease is consistent with successful treatment. It is worth noticing that most patients cleared BI, even those with very high BI at RFT without the need of a second round of twelve supervised doses, as suggested by Gonçalves et al [26].

Because of the small number of relapse cases in this cohort, the calculation of IR in different groups yielded a very low power. However, few studies have reported some associations. For example, high BI at diagnosis is commonly considered a risk factor for relapse [8], and Maghanoy *et al*. [12] observed the IR rise from 0.52 to 1/1000 py in a selected subset of 176 patients with high BI (≥ 4.0) prior to MB-MDT.

DG 2 is an indicator of a delayed diagnosis [5]. It reflects a longer presence and duration of growth of the bacteria in the body, thus it could increase the risk of relapse. Prabu et al [10] observed a high proportion of DG 2 in the patients diagnosed with relapse. In the present cohort, although two patients progressed towards deformity during relapse, none of them had DG 2 at RFT.

Two main pathways explain relapse in HD; it can be due to reactivation or reinfection. Persistent bacilli can be a source of reactivation [8]. On the other hand, in endemic regions where there is an ongoing transmission, re-infection may occur. *M leprae* molecular epidemiological studies and whole-genome sequencing methods have demonstrated that most relapses are, in fact, re-infections [28–29]. Preventing and detecting relapse remains a priority in endemic countries, as patients with relapse can be an important source of infection. Relapse is an indicator of the quality of HD control services [6] and is the main outcome for the measurement of the treatment efficacy. The low relapse rate after twelve-dose MB-MDT, such as presented in this paper, is clear evidence of the efficacy of fixed-dose MDT. National HD programs in endemic countries should urge physicians to endorse the twelve-dose MB scheme.

Although Brazil has the highest reported number of relapses [5] for all types of treatment schemes, the low incidence density demonstrated in this study is good evidence of the efficacy of twelve-dose MB-MDT. It is worth mentioning that ASA patients’ non-compliance rate is very low, as can be observed from the baseline data in which only 4.8% of the patients were excluded due to non-compliance. This fact, together with the absence of patients from our cohort in the linked dataset with the relapse cases registered in the national Hansen disease database, supports that no cases were lost to follow-up.

We recommend that the approaches to perform resistance surveillance and retesting of BI upon suspicion of relapse be maintained for monitoring of people affected by HD after RFT. Surveillance is also essential in highly endemic regions because of the risk of reinfection.
